# Ageism and Knowledge of Aging Among Future Healthcare Professionals

**DOI:** 10.1093/geroni/igaf122.3879

**Published:** 2025-12-31

**Authors:** Frances Hawes, ShuangShuang Wang, Inessah Cernohous

**Affiliations:** University of Wisconsin-Eau Claire, Eau Claire, Wisconsin, United States; Southwest Jiaotong University, Chengdu, Sichuan, China (People’s Republic); University of Wisconsin-Eau Claire, Eau Claire, Wisconsin, United States

## Abstract

As the global population ages, addressing ageist attitudes among future healthcare professionals is essential. This study surveyed 148 undergraduate students pursuing health-related careers at a large Midwestern university to assess their knowledge and attitudes about aging using the Ageism Attitude Scale (AAS) and Palmore’s Facts on Aging Quiz (FAQ II). Students demonstrated limited knowledge of aging but generally positive attitudes, with low negative ageism, moderate positive ageism, and some belief that aging restricts life. Regression analyses showed that attitudes varied by academic major, experience, and knowledge. Nursing majors reported higher positive ageism than pre-med students, and frequent contact with older adults was linked to lower restrictive views. Negative ageism was lowest among healthcare administration majors and students with formal training in aging. Aging knowledge was positively associated with more favorable attitudes and varied by school year, with sophomores and juniors scoring lower than freshmen.

